# Reduction of antibiotic prescriptions for acute respiratory tract infections in primary care: a systematic review

**DOI:** 10.1186/s13012-018-0732-y

**Published:** 2018-03-20

**Authors:** Anna Köchling, Christin Löffler, Stefan Reinsch, Anne Hornung, Femke Böhmer, Attila Altiner, Jean-François Chenot

**Affiliations:** 1 Clinic for Psychosomatic Medicine and Psychotherapy, University Medical Center, Rostock, Germany; 2 Institute of General Practice, University Medical Center, Rostock, Germany; 3 Department of Pediatric Pneumology, Immunology & Intensive Care Medicine Charité, University Medical Center Berlin, Berlin, Germany; 4 Institute for Biostatistics and Informatics in Medicine and Ageing Research, University Medical Center Rostock, Rostock, Germany; 5 Institute for Community Medicine—Department of Family Medicine, University Greifswald, Greifswald, Germany; 6 Institute of General Practice, University Medical Center, Rostock, Germany

**Keywords:** Acute respiratory tract infections, Antibiotics, Primary care

## Abstract

**Background:**

Although most respiratory tract infections (RTIs) are due to viral infections, they cause the majority of antibiotic (Abx) prescriptions in primary care. This systematic review summarises the evidence on the effectiveness of interventions in primary care aiming to reduce Abx prescriptions in patients ≥ 13 years for acute RTI.

**Methods:**

We searched the databases “MEDLINE/PubMed” and “Cochrane Library” for the period from January 1, 2005, to August 31, 2016, for randomised controlled trials (RCTs) in primary care aiming at the reduction of Abx prescriptions for patients suffering from RTI. Out of 690 search results, 67 publications were retrieved and 17 RCTs were included. We assumed an absolute change of 10% as minimal important change.

**Results:**

Twelve out of 17 included RCTs showed statistically significant lower Abx prescription rates in the intervention groups, but only six of them reported a clinically relevant reduction according to our definition. Communication skills training (CST) and point-of-care testing (POCT) were the most effective interventions. Pre-intervention Abx prescription rates varied between 13.5% and 80% and observed reductions ranged from 1.5 to 23.3%. Studies with post-intervention rates lower than 20% had no significant effects. Post-intervention observation periods ranged from 2 weeks up to 3.5 years. The design of the trials was heterogeneous precluding calculation of pooled effect size. The reporting of many RCTs was poor.

**Conclusions:**

CST and POCT alone or as adjunct can reduce antibiotic prescriptions for RTI. Eleven out of 17 trials were not successfully reducing Abx prescription rates according to our definition of minimal important change. However, five of them reported a statistically significant reduction. Trials with initially lower prescription rates were less likely to be successful. Future trials should investigate sustainability of intervention effects for a longer time period. The generalisability of findings was limited due to heterogeneous designs and outcome measures. Therefore, a consensus of designing and reporting of studies aiming at reducing antibiotic prescriptions is urgently needed to generate meaningful evidence.

## Introduction

Although most respiratory tract infections (RTIs) are due to viral infections, they cause the majority of antibiotic prescriptions in primary care [1]. Most patients suffering from RTI do not benefit from an antibiotic treatment since severity and duration of the disease are not relevantly altered. On the contrary, many patients experience side effects such as diarrhoea and rash [2]. Additionally, unnecessary antibiotic prescriptions contribute to increasing bacterial resistance to standard antibiotics [3].

Various interventions have been evaluated to reduce antibiotic prescribing for RTI, e.g. public campaigns, distribution of printed educational material or group education meetings [4–6]. A recent systematic review found moderate short-term effects on antibiotic prescribing of interventions facilitating shared decision-making [7]. Another global review summarised the effects of antimicrobial stewardship programs in ambulatory care including interventions for all infectious conditions and children. This review found low-strength evidence for interventions including provider and/or patient education, guidelines, delayed prescribing (DP) and computerised clinical decision support systems (CDSS) [8]. It is expected that within the next years, antibiotic stewardship programs will need to be established worldwide [9]. So far, there is still a discussion and uncertainty about which specific elements of interventions lead to high effectiveness and sustainability [10]. Therefore, this review aims to update and summarise current evidence of various interventions in primary care on reducing antibiotic prescription rates (Abx prescription rates) due to acute RTIs in patients ≥ 13 years.

## Methods

This is a systematic review reported according to the PRISMA Statement [11]. This systematic review was not registered.

### Search methods for identification of trials

The systematic literature search was carried out in MEDLINE/PubMed and the Cochrane Library using the following search terms:

((antibiotic*) AND (“respiratory tract infection” OR “respiratory tract infections” OR “res-piratory infection”) AND (communication OR training OR “point of care test” OR “rapid strep test” OR “delayed prescribing” OR intervention* OR “electronic decision support” OR “clinical decision support system” OR “clinical decision support systems” OR “shared decision making”) AND (“primary care” OR “primary health care” OR “medical care”)) OR ((“Anti-Bacterial Agents”[Mesh]) AND (“Respiratory Tract Infections”[Mesh]) AND (“Primary Health Care”[Mesh] OR “Physicians, Primary Care”[Mesh]))

Additionally, the bibliographies of the included trials were screened for relevant intervention trials. We could not search further databases like EMBASE due to limited access and lack of funding.

### Inclusion and exclusion criteria

The literature search included (cluster-) randomised controlled trials (RCTs) investigating the effect of interventions aiming to reduce antibiotic prescriptions for RTIs for patients ≥ 13 years in primary care settings. We excluded pilot trials and non-randomised trials. Eligible interventions were educational seminars, feedback on prescribing behaviour, patient education, communication skills training (CST) for physicians and diagnostic tools such as point-of-care tests (POCT) or (electronic) CDSS. We did not take public campaigns into account. These address a broad audience and use mass media to raise awareness for the problem of inadequate antibiotic prescribing and its influence on bacterial resistance. As our systematic review focuses on primary care, this type of intervention is not considered.

We investigated the primary outcome of Abx prescription rate as well as the number of antibiotic prescriptions for acute upper/lower RTIs (cough and sore throat). Any reported secondary outcome such as patient’s reconsultation rate, days to recovery from RTI, prescribed class of antibiotic, rate of inappropriate antibiotic prescriptions (prescriptions not according to guidelines), Abx prescription rate for specific RTIs or usage of diagnostic devices was of interest to this systematic review.

We only included patients ≥ 13 years as we know that clinical decision-making in paediatric medicine differs from adults. Further, the communication in dyadic consultations between adults differs from “doctor-parent-child triads” and requests other communication styles [12]. In consequence, we excluded children under 13 years. We included primary care physicians working in ambulatory care. Due to different health systems, we included physicians working in practices or primary care clinics.

We only considered publications written in English, German or French dated January 2005 till July 2016.

### Trial selection

Two reviewers (AK, SR or JFC) independently reviewed the titles, index terms and abstracts of the identified references and rated each paper as potentially relevant or not. Discrepancies were resolved by consensus.

### Data extraction

Relevant information for each trial included into the review was extracted by one reviewer and controlled independently by another one (AK, SR). Discrepancies were resolved by consensus. Three authors had to be contacted due to missing or unclear data about the age of included patients [13–15]. Gjelstad et al. offered odds ratios (OR) for antibiotic prescriptions for the age group ≥ 13 years, but no Abx prescription rate. It was possible to get unpublished Abx prescription rates for the subgroup of patients ≥ 13 years [14]. Missing data—like *p* values or absolute numbers of antibiotic prescriptions—was indicated as “not specified” in our tabular summaries of included RCTs (see Tables 1 and 2).

**Table 1:** Antibiotic prescription rate of trials with baseline data and post-intervention measurements

Study	Absolute number of prescribed Abx (in %/95% CI/*p* value) for IG and GC; adjusted OR; RR	Difference in Abx prescription rates between corresponding study arms (in %)/difference in differences for Abx prescriptions between IG and CG	Odds ratio for Abx prescriptions (95% CI, *p* value)	Absolute reduction of Abx prescriptions in the corresponding study arm (in %)
Bjerrum et al. 2006 Spain	T0: IG: n.s. (36%/29–44%/n.s.) CG: not performed T1: IG: n.s. (24%/20–29%/n.s.) CG: n.s. (32%/27–38%/n.s.)	T0: n.s. T1: Δ (IG − CG) = − 12% Difference in differences: n.s.	T0: n.s. T1: IG and CG: 0.67	IG: Δ (T1 − T0) = − 12% CG: n.s.
Altiner et al. 2007 Germany	T0: IG: n.s. (36.4%/n.s./n.s.) CG: n.s. (54.7%/n.s./n.s.) T1: IG: n.s. (29.4%/n.s./n.s.) Adjusted OR for IG: 0.58 (95% CI 0.43–0.78, *p* < 0.001) CG: n.s. (59.4%/n.s./n.s.) Adjusted OR for CG: 1.52 (95% CI 1.19–1.95, *p* = 0.001) T2: IG: n.s. (36.7%/n.s./n.s.) Adjusted OR for IG: 0.72 (95% CI 0.54–0.97, *p* = 0.028) CG: n.s. (64.8%/n.s./n.s.) Adjusted OR for CG: 1.31 (95% CI 1.01–1.71, *p* = 0.044)	T0: Δ (IG − CG) = − 18.3% T1: Δ (IG − CG) = − 30% T2: Δ (IG − CG) = − 28.1% Difference in differences: IG for T2: − 9.8%	T0: IG and CG: 0.47 T1: IG and CG: 0.28 T2: IG and CG: 0.31	IG: Δ (T1 − T0) = − 7% Δ (T2 − T1) = − 7.3% Δ (T2 − T0) = + 0.3% CG: Δ (T1 − T0) = + 4.7% Δ (T2 − T1) = + 5.4% Δ (T2 − T0) = + 10.1%
Gonzales et al. 2013 USA	T0: IG 1: n.s. (80%/n.s./n.s.) IG 2: n.s. (74%/n.s./n.s.) CG: n.s. (72.5%/n.s./n.s.) T1: IG 1: n.s. (68.3%/n.s./n.s.) IG 2: n.s. (60.7%/n.s./n.s.) CG: n.s. (74.3%/n.s./n.s.)	T0: Δ (IG 2 − IG 1) = − 6% Δ (IG 1 − CG) = + 7.5% Δ (IG 2 − CG) = + 1.5% T1: Δ (IG 2 − IG 1) = − 7.6% (*p* = 0.67) Δ (IG 1 − CG) = − 6% (*p* = 0.003) Δ (IG 2 − CG) = − 13.6% (*p* = 0.01) Difference in differences: For IG 1: − 13.5% For IG 2: − 15.1%	T0: IG 1 and IG 2: 1.4 IG 1 and CG: 1.52 IG 2 and CG: 1.08 T1: IG 1 and IG 2: 1.39 IG 1 and CG: 0.75 IG 2 and CG: 0.53	I 1: Δ (T1 − T0) = − 11.7% I 2: Δ (T1 − T0) = − 13.3% C: Δ (T1 − T0) = + 1.8%
Gjelstad et al. 2013* Norway	T0: IG: n.s. (34.3%/31.8–36.9/n.s.) CG: n.s. (35.2%/CI 32.8–37.7/n.s.) T1: IG: n.s. (32.8%/30.3–35.3/n.s.) CG: n.s. (36.9%/34.2–39.7/n.s.)	T0: Δ (IG − CG) = − 0.9% T1: Δ (IG − CG) = − 4.1% Difference in differences: IG: + 0.2%	T0: IG and CG: 0.96 T1: IG and CG: 0.83	IG: Δ (T1 − T0) = − 1.52% (95% CI − 2.85 to − 0.18, *p* = 0.027) CG: Δ (T1 − T0) = + 1.70 (95% CI 0.69–2.72, *p* = 0.002)
Andreeva et al. 2014 Russia	Subgroup of 13 physicians: T0: IG: 28/47 (59%/n.s./n.s.) CG: 21/34 (62%/n.s./n.s.) T1: IG: 30/81 (37%/n.s./n.s.) CG: 44/62 (71%/n.s./n.s.) T1 for all 18 GPs: IG: n.s. (37.6%/n.s./n.s.) CG: n.s. (58.9%/n.s./n.s.) T2 for all 18 GPs: IG: n.s. (40.6%/n.s./n.s.) CG: n.s. (71.8%/n.s./n.s.)	T0: Δ (IG − CG) = − 3% T1 for subgroup of 13 GPs who also participated in baseline study: Δ (IG − CG) = − 34% T1 for all 18 GPs: Δ (IG − CG) = − 21.3% (*p* = 0.006) T2 for all 18 GPs: Δ (IG − CG) = − 31.2% (*p* = 0.0001) Difference in differences: n.s.	T0 only for 13 GPs: IG and CG: 0.91 T1 for subgroup of 13 GPs who also participated in baseline study: IG and CG: 0.24 T1 for all 18 GPs: IG and CG: 0.42 T2 for all 18 GPs: IG and CG: 0.27	IG (subgroup of 13 GPs): Δ (T1 − T0) = − 22% CG (subgroup of 13 GPs): Δ (T1 − T0) = + 9% IG (all GPs): Δ (T2 − T1) = + 4% CG (all GPs): Δ (T2 − T1) = + 12.9%
Gulliford et al. 2014 UK	T0: IG: n.s. (53%/n.s./n.s.) CG: n.s. (52%/n.s./n.s.) T1: IG: n.s. (52%/n.s./n.s.) CG: n.s. (52%/n.s./n.s.)	T0: Δ (IG − CG) = + 1% T1: Δ (IG − CG) = 0% Adjusted mean difference (adjusted for pre-intervention value, as well as mean age and proportion of women at each practice): − 1.85% (95% CI 0.10–3.59%; *p* = 0.038) Difference in differences: IG: − 1%	T0: IG and CG: 1.04 T1: IG and CG: 1.00	IG: Δ (T1 − T0) = − 1% CG: Δ (T1 − T0) = 0%
Little et al. 2013 Belgium, Spain, Wales, Great Britain, Poland, Netherlands	T0: 3742/6771 (55.3%/n.s./n.s.) T1: Abx prescription rates regarding study arms: CG: 508/870 (58%/n.s./n.s.), OR = 1.00 Internet-based training for CRP-POCT: 368/1062 (35%/n.s./n.s.) OR = 0.54 (95% CI 0.40–0.68; *p* < 0.001) Internet-based CST: 476/1170 (41%/n.s./n.s.) OR = 0.69 (95% CI 0.54–0.85; *p* < 0.001) Internet-based CST + CRP-POCT: 366/1162 (32%/n.s./n.s.) OR = 0.46 (95% CI 0.35–0.60; *p* < 0.001) Abx prescription rates regarding factorial groups: Cumulative non-CRP-training group: 984/2040 (48%/n.s./n.s.) Cumulative CRP-training group: 734/2224 (33%/n.s./n.s.) Cumulative non-CST group: 876/1932 (45%/n.s./n.s.) Cumulative CST group: 842/2332 (36%/n.s./n.s.)	T1: Δ (cumulative CRP group − cumulative non-CRP group): − 15% Δ (cumulative CST group − cumulative non-CST group): − 9% Difference in differences: n.s.	T0: n.s. T1: Cumulative CRP-training group and cumulative non-CRP training group: 0.54 (95% CI 0.42–0.69, *p* < 0.0001) Cumulative CRP-training group and cumulative communication training group: 0.88 Cumulative communication training group and cumulative non-communication training group: 0.69 (95% CI 0.54–0.87, *p* < 0.0001)	CG: Δ (T1T0) = + 3% Internet-based training for CRP-POCT: Δ (T1 − T0) = − 20% Internet-based communication training: Δ (T1 − T0) = − 14% Internet-based communication training + CRP-POCT: Δ (T1 − T0) = − 23% Cumulative non-CRP-training group: Δ (T1 − T0) = − 7% Cumulative CRP-training group: Δ (T1 − T0) = − 22% Cumulative no-CST group: Δ (T1 − T0) = − 10% Cumulative CST group: Δ (T1 − T0) = − 19%
Meeker et al. 2016 USA	T0 for each study group: IG 1: 1057/2132 (49.6%/47.5–51.7/n.s.) IG 2: 497/1491 (33.3%/30.9–37.7/n.s.) IG 3: 433/1236 (35.0%/32.4–37.7/n.s.) IG 1 + 2: 702/1977 (35.5%/33.4–37.6/n.s.) IG 1 + 3: 368/1511 (24.4%/22.2–26.5/n.s.) IG 2 + 3: 782/2362 (33.1%/31.2–35.0/n.s.) IG 1 + 2 + 3: 558/2178 (25.6%/23.8–37.5/n.s.) CG: 692/1866 (37.1%/34.9–39.3/n.s.) T1 for each study group: IG 1: 722/2388 (30.2%/28.4–32.1/n.s.) IG 2: 324/1979 (16.4%/14.7–18.0/n.s.) IG 3: 311/1620 (19.2%/17.3–21.1/n.s.) IG 1 + 2: 341/2131 (16.0%/14.5–17.6/n.s.) IG 1 + 3: 139/2014 (6.9%/5.8–8.0/n.s.) IG 2 + 3: 340/2240 (15.2%/13.7–16.7/n.s.) IG 1 + 2 + 3: 249/2492 (10.0%/8.8–11.2/n.s.) CG: 502/2095 (24.0%/22.1–25.8/n.s.)	T0: Δ (IG 1 − CG): + 12.5% Δ (IG 2 − CG): − 3.8% Δ (IG 3 − CG): − 2.1% Δ (IG 1 − IG 2): + 16.3% Δ (IG 1 − IG 3): + 14.6% Δ (IG 2 − IG 3): − 1.7% T1: Δ (IG 1 − CG): + 6.2% Δ (IG 2 − CG): − 7.6% Δ (IG 3 − CG): − 4.8% Δ (IG 1 − IG 2): + 13.8% Δ (IG 1 − IG 3): + 11% Δ (IG 2 − IG 3): − 2.8% Difference in differences: IG 1: − 6.3% IG 2: − 3.8% IG 3: − 2.7% IG 1 + 2: − 6.4% IG 1 + 3: − 4.4% IG 2 + 3: − 4.8% IG 1 + 2 + 3: − 2.5%	T0: IG 1 and CG: 1.67 IG 2 and CG: 0.85 IG 3 and CG: 0.91 IG 1 and IG2: 1.97 IG 1 and IG 3: 1.83 IG 2 and IG 3: 0.93 T1: IG 1 and CG: 1.37 IG 2 and CG: 0.62 IG 3 and CG: 0.75 IG 1 and IG 2: 2.21 IG 1 and IG 3: 1.82 IG 2 and IG 3: 0.83	IG 1: Δ (T1 − T0) = − 19.4% IG 2: Δ (T1 − T0) = − 16.9% IG 3: Δ (T1 − T0) = − 15.8% IG 1 + 2: Δ (T1 − T0) = − 19.5% IG 1 + 3: Δ (T1 − T0) = − 17.5% IG 2 + 3: Δ (T1 − T0) = − 17.9% IG 1 + 2 + 3: Δ (T1 − T0) = − 15.6% CG: Δ (T1 − T0) = − 13.1% Adjusted analysis (hierarchical regression model) for factorial study groups: CG: Δ (T1 − T0) = − 11.0% IG 1: Δ (T1 − T0) = − 16% IG 2: Δ (T1 − T0) = − 18.1% IG 3: Δ (T1 − T0) = − 16.3%

*n.s.* not specified, *IG* intervention group, *CG* control group, *RR* relative risk, *OR* odds ratio, *POCT* point-of-care testing, *CRP* C-reactive protein, *CDSS* clinical decision support system, *95% CI* 95% confidence interval

*Unpublished data for patient sample ≥ 13 years, provided by Gjelstad et al.

**Table 2:** Abx prescription rates of trials with one post-intervention measurement

Study	Absolute number of prescribed Abx (in %/95% CI/*p* value) for IG and CG; adjusted OR; RR	Difference in Abx prescription rates between corresponding study arms (in %)	Odds ratio for Abx prescriptions (95% CI; *p* value)	Absolute reduction of Abx prescriptions in the corresponding study arm (in %)
Briel et al. 2006 Switzerland	T1: IG 1: 46/293 (15.7%/n.s./n.s.) Adjusted OR: 0.86 (95% CI 0.4–1.93) IG 2: 35/259 (13.5%/n.s./n.s.) CG: 61/285 (21.4%/n.s./n.s.)	T1: Δ (IG 2 − IG 1) = − 2.2% Δ (IG 1 − CG) = − 5.7% Δ (IG 2 − CG) = − 7.9%	T1: IG 1 and CG: 0.68 IG 2 and CG: 0.57 IG 1 and IG 2: 1.19	n.s.
Linder et al. 2009 [25] USA	T0: unpublished data T1: IG: 4601/11954 (39%/n.s./n.s.) CG: 4316/10007 (43%/n.s./n.s.)	T1: Δ (IG − CG) = − 4%	T1: IG and CG: 0.83 (95% CI 0.6–1.2; *p* = 0.30)	n.s.
Cals et al. 2009* Netherlands	Abx prescription rates regarding study arms (only for T1): POCT group: 43/110 (39%/25.6–52.6/n.s.) CST group: 28/84 (33%/19.5–47.1/n.s.) POCT + CST-group: 27/117 (23%/11.6–34.6**/**n.s.) Usual care: 80/120 (67%/53.9–79.5/n.s.) Abx prescription rates regarding factorial groups (T1 ± T2): POCT group: T1: 70/227 (30.8%/21.8–39.8/n.s.) T2: 102/227 (44.9%/35.2–54.6/n.s.) CG for POCT = no POCT group: T1: 108/204 (52.9%/43.0–62.8/n.s.) T2: 119/204 (58.3%/48.5–68.1/n.s.) Comparison of the 2 groups: T1: *p* < 0.02 T2: *p* < 0.01 CST group: T1: 55/201 (27.4%/25.6–36.6/n.s.) T2: 76/201 (37.8%/28.1–47.5/n.s.) CG for CST = no CST group: T1: 123/230 (53.5%/43.8–63.2/n.s.) T2: 145/230 (63%/53.6–72.4/n.s.) Comparison of the 2 groups: T1: *p* < 0.01 T2: *p* < 0.001	T1: Δ (POCT group − CG) = − 28% Δ (CST group − CG) = − 34% Δ (POCT/CST − CG) = − 44% Δ (POCT − CST) = + 6% Δ (POCT − POCT/CST) = + 16% Δ (CST − POCT/CST) = + 10% Δ (CST − POCT) = − 6% Δ (POCT/CS − TPOCT) = − 16% Δ (POCT/CST − CST) = − 10% Only calculation for T2* Δ (cumulative POCT group − cumulative non-POCT group): − 13.4% Δ (cumulative CST group − cumulative non-CST group): − 25.2%	T1: Cumulative POCT group and cumulative non-POCT group: 0.39 Cumulative POCT group and cumulative CST group: 1.18 Cumulative CST group and cumulative non-CST group: 0.33 T2: Cumulative POCT group and cumulative non-POCT group: 0.58 Cumulative POCT group and cumulative CST group: 1.34 Cumulative CST group and cumulative non-CST group: 0.36	n.s.
Cals et al. 2010* Netherlands	T1: IG: 56/129 (43.4%/n.s./n.s.) CG: 73/129 (56.6%/n.s./n.s.) RR = 0.77 (95% CI 0.56–0.98) T2: IG: 68/129 (52.7%/n.s./n.s.) CG: 84/129 (65.1%/n.s./n.s.) RR = 0.81 (95% CI 0.62–0.99)	Only calculation for T2 T2: Δ (IG − CG) = − 12.4%	T1: IG and CG: 0.59 T2: IG and CG: 0.6	n.s.
Linder et al. 2010 USA	T0: baseline (unpublished data) T1: IG: 3912/8406 (47%/n.s./n.s.) CG: 4761/10082 (47%/n.s./n.s.)	T1: Δ (IG − CG) = 0%	T1: IG and CG: 0.97 (95% CI 0.7–1.4, *p* = 0.87)	n.s.
Worrall et al. 2010 Canada	T1: IG: 33/75 (43.2%/n.s./n.s.) CG: 32/74 (44%/n.s./n.s.) *p* = 0.924	T1: Δ (IG − CG) = − 0.8%	T1: IG and CG: 0.97	n.s.
Llor et al. 2011 Spain	T1: IG: 123/281 (43.8%/n.s./*p* < 0.001) CG: 168/262 (64.1%/n.s./n.s.)	T1: Δ (IG − CG) = − 20.3%	T1: IG and CG: 0.46	n.s.
McGinn et al. 2013 USA	T1: IG: 171/586 (29.2%/n.s./n.s.) CG: 153/398 (38.4%/n.s./n.s.) Comparison IG/CG: RR 0.73 (95% CI 0.58–0.92, *p* = 0.008) age-adjusted RR 0.74 (95% CI 0.60–0.92; *p* = 0.008)	T1: Δ (IG − CG) = − 9.2%	T1: IG and CG: 0.66	n.s.
Hui Min Lee et al. 2016 Singapore	T1: IG: 94/457 (20.6%/n.s./n.s.) CG: 81/457 (17.7%/n.s./n.s.)	T1: Δ (IG − CG) = + 2.9%	T1: IG and CG: 1.20 (95% CI 0.84–1.72, *p* = 0.313)	n.s.

*CRP* C-reactive protein, *CDSS* clinical decision support system, *95% CI* 95% confidence interval, *CST* communication skills training, *CG* control group, *n.s.* not specified, *IG* intervention group, *RR* relative risk, *OR* odds ratio, *POCT* point-of-care testing

*Cals et al. 2009 and Cals et al. 2010: Antibiotic prescription data of the patient sample were captured during the index consultation (T1) and within 28 days after the index consultation. As T2 prescription rates include T1 prescription rates, this study is considered as a study with one post-intervention measurement

### Summary of Abx prescription rates for acute upper/lower RTI

If available, antibiotic use was presented as the absolute number of prescribed antibiotics. In addition, relative Abx prescription rates expressed as a percentage—with the corresponding 95% confidence intervals (= 95% CI) and *p* values—were indicated as well as the difference of Abx prescription rates between intervention group (IG) and control group (CG) (in percent, OR, relative risks (RR) (Tables 1 and 2). The Abx prescription rates before (T0) and after the intervention (T1 or T2) were shown for trials providing pre-post comparison. As RCTs were heterogeneous in study design and therefore in time points for T1 or T2, we decided to not exclude RCTs by defining a binding point of time for T1 or T2. Details of included RCTs can be seen in a tabular summary of study characteristics.

### Assessment of risk of bias

**Fig. 1:**
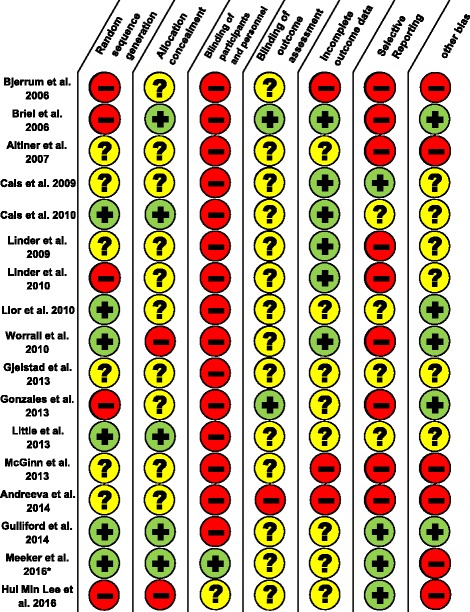
Assessment of risk of bias. Legend: *plus data from published study protocol.  low risk of bias.  high risk of bias.  unclear risk of bias

Two researchers (AK with SR, FB or JFC) independently assessed the risk of bias using the *Cochrane Collaboration’s tool for assessing risk of bias in randomised trials* (Fig. 1) [16]. We discussed and resolved discrepancies by consensus*.*

### Evaluation of intervention effect

Due to the heterogeneity of study designs, outcome measures and Abx prescription rates, we considered a pooled estimate of the effect size with Cohen’s *d* to be inappropriate [17].

There is no consensus on which change in Abx prescribing rates marks a meaningful intervention effect. The difference between current Abx prescription rate and optimal prescription rate reflects the range for reducing antibiotic prescriptions to a meaningful extent. But the generalisability for determining an optimal rate is limited by patient age and condition with different likelihoods for bacterial genesis (pneumonia vs. common cold) [18]. One report from the year 2016 states that 44% of all ambulatory antibiotic prescriptions in the USA are due to RTIs. It is said that 50% of these prescriptions are inappropriate leading to an optimal prescribing rate of about 20% for all RTIs [18]. Another publication suggests a prescribing rate of 10–15% for acute cough [19].

As a compromise in this complex field and to simplify the overview of clinically relevant reductions in Abx prescriptions, we will consider an absolute difference of 10% between IG and CG for studies with post-intervention as minimal important change. For studies with baseline and follow-up, we regard a difference in differences of 10% as minimal important change. We acknowledge that this threshold is arbitrary, but a different threshold would not have changed our findings fundamentally.

## Results

### Study selection

**Fig. 2:**
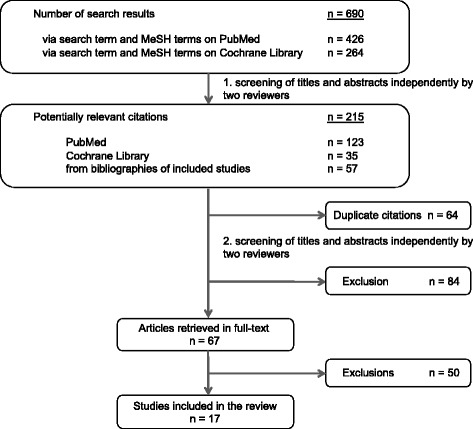
Process of trial selection. MeSH - medical subject heading

We searched databases on the 31st of July in 2016 and identified 690 publications. Reviewers independently screened for potentially relevant publications and categorised 215 publications as potentially relevant. After removal of duplicates (*n* = 84), the remaining 151 titles and abstracts were screened for eligibility and discussed by the reviewers. Disagreements between reviewers were resolved by consensus. Major reasons for exclusion were different trial populations, e.g. including children, trials carried out in non-primary care settings and non-randomised study designs. We excluded 84 out of 151 publications. A total of 67 potentially relevant articles were fully screened for inclusion and exclusion criteria (Fig. 2).

Seventeen trials were finally included in this analysis.

### Description of included trials

Seventeen RCTs met the inclusion criteria and were included in this review (Tables 3 and 4). Thirteen trials were cluster RCTs based on physician-, practice- or educational group levels as clusters [13–15, 20–29]. Four RCTs were randomised at patient level [30–33]. The majority of trials used a two-arm study design [13–15, 20, 24, 25, 27, 28, 30–33]. The remaining trials employed a three-arm [21, 23] or factorial study design [22, 26, 29].

**Table 3:** Characteristics of studies with baseline data and post-intervention measurements

Study, country and study design	Inclusion criteria for patients	Number of patients (I/C)*; average age of patients (I/C)*; number of physicians (I/C*)	Intervention and control group*	Data collection periods	Number of adverse events (e.g. hospitalisations, deaths) or side effects
Bjerrum et al. 2006 Spain Two-armed cluster-randomised controlled intervention study	> 14 years Consultation due to upper/lower RTI	T0: 1114/not performed; n.r. T1: 1674/2462; n.r. Physicians on T0: 52 Physicians on T1: 17/35	IG: training for physicians + feedback and reflection of baseline—Abx prescription rate + introduction to POCT (CRP and RADT) CG: care as usual	Abx prescription rate right after initial consultation: T0: only for IG (Dec.–Feb. 02/03) T1: 1 year after I (Dec.–Feb. 04/05)	n.r.
Altiner et al. 2007 Germany Two-armed cluster-randomised controlled intervention study	> 16 years First episode of acute cough (no cough for the previous 8 weeks)	T0: 753/898; 42.2/42 T1: 675/885; 44.9/43.9 T2: 787/920; 41.7/41.8 Physicians on T0: 104 Physicians on T1: 42/44 Physicians on T2: 28/33	IG: communication training for physicians + handouts for patients and waiting room poster CG: care as usual	Abx prescription rate right after initial consultation: T0: Nov.–Jan. 03/04 T1: 6 weeks after I (Feb.–Apr. 2004) T2: 1 year after I (Jan.–Mar. 2005)	n.r.
Gonzales et al. 2013 USA Three-armed cluster-randomised controlled intervention study	13–64 years Consultation due to uncomplicated acute bronchitis (ICD-9-CM code 466.0; ICD-9-CM code 490) (No RTI consultation in the last 30 days)	T0: Arm 1: 3639; n.r. Arm 2: 2974; n.r. Arm 3: 3195; n.r. T1: Arm 1: 1001; n.r. Arm 2: 1017; n.r. Arm 3: 950; n.r. 11 “practice centres” for each study arm Arm 1: 68 physicians Arm 2: 41 physicians Arm 3: 46 physicians	IG 1: 1) Training for physicians (via “clinical champion”) 2) Patient brochure 3) Examination room poster with clinical algorithm for acute bronchitis IG 2: 1) and 2) instead of 3) ➔ clinical algorithm integrated in practice software CG: care as usual	Abx prescription rate right after initial consultation: T0: baseline (3 successive winter periods from Oct. to Mar. 2007–2009) T1: Oct. 2009–Mar. 2010	Hospitalisations and emergency room visits occurred rarely in all study arms (no published data)
Gjelstad et al. 2013** Norway Two-armed cluster-randomised controlled intervention study	> 13 years Consultation due to acute RTI	Number of RTI consultations on T0 for patients > 13 years: 43880/46518 Number of RTI-consultations on T1 for patients > 13 years: 47522/47868 Number of physicians on T0: 39 education groups (202 physicians); n.r./41 education groups (232 physicians); n.r. Number of physicians on T1: 39 education groups (183 physicians); 48.3/40 education groups (199 physicians); 49.7	IG 1: 1) Meeting: guidelines for RTI and strategy of delayed prescribing; installation of additional program for 2 of 4 practice software (reminders to document the number of days for delayed prescribing) 2) Meeting: feedback on prescribing rates based on individual data 3) 1-day seminar: reinforcement of intervention IG 2: Training regarding adequate pharmacotherapy on patients > 70 years in 2 group meetings and 1-day peer visit, based on individual prescribing rates; antibiotic therapy was not content	2 data collection periods for Abx prescription rate: T0: (Abx prescription rate during 1 year before I by retrospective data analysis with special software) I: Dec. 2005–May 2006 T1: Abx prescription rate during 1 year (after I)	n.r.
Little et al. 2013 Belgium, Spain, Wales, Great Britain, Poland, Netherlands Multinational cluster-randomised controlled intervention study, factorial study design	> 18 years First episode of acute cough (max. of 28 days) First episode of upper/lower RTI	T0: 6771; 49.6 T1: 4264 Arm 1 **=** CG: 870 Arm 2 **=** IG 1: 1062 Arm 3 **=** IG 2: 1170 Arm 4 **=** IG 3: 1162 Factorial groups: POCT-CRP-group (arms 2 + 4): 2224; 51.0 CG for CRP-POCT = no CRP-POCT group (arms 1 + 3): 2040; 50.9 CST-group (arms 3 + 4): 2332; 51.1 CG for CST = no CST group (arms 1 + 2): 1932; 50.8 Physicians on T0: 259 practices Physicians on T1: 228 practices; 372 physicians	CG: care as usual IG 1: Internet-based training for CRP-POCT IG 2: Internet-based communication training IG 3: Internet-based training for communication training + CRP-POCT	Abx prescription rate right after initial consultation: T0: Oct.–Dec. 2010 T1: Feb.–May 2011	Deaths: none Hospitalisations: CG: 2 IG 1: 10 IG 2: 6 IG 3: 12 Difference in hospitalisation rate between cumulative CRP group and cumulative non-CRP group: 22/8 (OR* = 2.91, 95% CI 0.96–8.85, *p* = 0.06) Cluster-adjusted: OR* = 2·61, 95% CI 1.07–6.35, *p* = 0.034
Andreeva et al. 2014 Russia Two-armed cluster-randomised controlled intervention study	Patients ≥ 18 years with first episode of acute cough/lower RTI (including acute bronchitis, pneumonia and infectious exacerbations of COPD or asthma)	T0 for subgroup of 13 GPs: 47/34; n.r. T1 for subgroup of 13 GPs: 81/62; n.r. T1: 101/78; 50.8/50.8 Physicians: a total of 18 GPs participated, but only 13 GPs participated in baseline and intervention period	IG: 1) Registration of patient’s symptoms, clinical examination and therapy in electronic case report form 2) 2 training sessions concerning the CRP test, guidelines about the interpretation of CRP results, discussion about paper cases of patients with different RTIs and different CRP values CG: 1)	Abx prescription rate during I period (12 weeks—from 30 January to 30 April 2010) T0: only for 13 GPs 2 months before data collection T1: Abx prescription rate right after initial consultation T2: Abx prescription rate 2 weeks after initial consultation	n.r.
Gulliford et al. 2014 UK Two-armed cluster-randomised controlled intervention study	Patients aged 18–59 years consulting for RTI	T0: 292,398/264,137; n.r. T1: 294,929/263,895; n.r. 50 family practices were allocated to IG, and 50 practices were allocated to CG	IG: decision support tool including a summary of antibiotic prescribing recommendations and research evidence concerning no antibiotic or delayed antibiotic prescribing strategies + a single-sided patient information sheet, information on the definite indications for antibiotic prescription CG: care as usual	Abx prescription rate during two 12-month data collection periods: T0: 12-month period before I T1: 12-month period after I	n.r.
Meeker et al. 2016 USA Cluster-randomised controlled intervention study, 2 × 2 × 2 factorial design	Patients ≥ 18 years with no visit for acute RTI within the prior 30 days	No. of patients: 14,753/16,959 No. of patients T0/T1 for each study group: IG 1: 2132/2388 IG 2: 1491/1979 IG 3: 1236/1620 IG 1 + 2: 1977/2131 IG 1 + 3: 1511/2014 IG 2 + 3: 2362/2240 IG 1 + 2 + 3: 2178/2492 CG: 1866/2095 No. of practices/physicians for T0 and T1: 47/248 No. of practices/physicians/mean age of patients for each study group: IG 1: 6/42/48 IG 2: 7/35/53 IG 3: 4/20/47 IG 1 + 2: 6/34/50 IG 1 + 3: 6/35/48 IG 2 + 3: 6/27/49 IG 1 + 2 + 3: 6/28/43 CG: 6/27/49	7 IGs: I1: “Suggested alternatives”—EHR-based intervention (resembling CDSS) triggered by a RTI. User gets a list of alternative treatment (e.g. over-the-counter medicine) I2: “Accountable justification”—EHR-based I triggered by antibiotic prescriptions. User must explicitly justify the treatment decision. I3: “Peer comparison”—monthly e-mail with individual number and proportion of adequate and inadequate antibiotic prescriptions for acute RTI compared with other physicians I4: I1 + I2 I5: I1 + I3 I6: I2 + I3 I7: I1 + I2 + I3 CG: care as usual	Abx prescription rate for inadequate antibiotic prescriptions for acute RTI T0: 18-month baseline for each practice T1: 18-month period (beginning Nov. 2011 to Oct. 2012, ending for the last practice in Apr. 2014)	Return visit rate within 30 days after initial consultation in which no antibiotics were prescribed: Among CG practices: 0.43% (95% CI 0.25–0.70) Among I2 and I3 practices: 1.41% (95% CI 1.06–1.85, *p* < 0.001) Among return visits, a random sample of 33 cases across all study groups was monitored for complications/hospitalizations: 11 cases of pneumonia, 1 otitis media, 1 pneumonia + otitis media 1 hospitalisation for pneumonia

*Abx prescription rate antibiotic prescription rate, *CRP* C-reactive protein, *CDSS* clinical decision support systems, *CG* control group, *EHR* electronic health record, *IG* intervention group, *n*.*r*. not reported, *POCT* point-of-care-testing, *RADT* rapid antigen detection test, *RTI* respiratory tract infection

**Unpublished data for patient sample ≥ 13 years, provided by Gjelstad et al.

**Table 4:** Characteristics of trials with one post-intervention measurement

Study, country and study design	Inclusion criteria for patients	Number of patients (I/C)*; average age of patients (I/C)*; number of physicians (I/C*)	Intervention and control group*	Data collection periods	Number of adverse events (e.g. hospitalisations, deaths) or side effects
Briel et al. 2006 Switzerland Three-armed cluster-randomised controlled intervention study	> 18 years First episode of acute RTI (symptoms for max. of 28 days)	T1: “Unlimited” IG: 293; 43.6 “Full” IG: 259; 41.4 CG: 285; 40.5 15 Physicians in each study group in total: 45 physicians in 45 practices	“Limited” IG: guidelines on RTI “full” IG: additional 6-h seminar on patient-centred communication + 2-h telephone feedback CG: (care as usual)	Abx prescription rate during 2 weeks after initial consultation, registered by study pharmacies T1: January–May 2004	Number of deaths and hospitalisations: “Limited” IG: 1 death “Full” IG: 3 hospitalisations CG: none
Cals et al. 2009** Netherlands Cluster-randomised controlled intervention study, factorial design	> 18 years Acute cough due to lower RTI (max. of 4 weeks)	T1 and T2: Arm 1: 110 Arm 2: 84 Arm 3: 117 Arm 4: 120 Factorial groups: POCT-group (arm 1 + arm 3): 227; 49.4 CG for POCT = no POCT group (arm 2 + arm 4): 204; 50.3 CST group (arms 2 + 3): 201; 51.4 CG for CST = no CST group (arms 1 + 4): 230; 48.5; 10 physicians (5 practices) in each study arm on T1 and T2	IG 1: POCT (CRP) IG 2: CST IG 3: POCT+CST CG: care as usual	Data collection in two winter periods 2005/2006 and 2006/2007: T1: after initial consultation T2: 28 days after initial consultation	No hospitalisations and deaths during the study
Linder et al. 2009 USA Two-armed cluster-randomised controlled intervention study	Consultation due to upper/lower acute RTI	Number of consultations due to RTI: T1: 11954/10007; 48/49 Physicians on T1: 262/181 (in 27 “practice centres”)	IG: “ARI Smart Form” = CDSS Collection of patient data, documentation of diagnosis and therapy, presentation of therapy options with integrated CDSS Print-out option for patient handouts and specialised literature CG: care as usual	Abx prescription rate right after initial consultation: T0: baseline T1: Abx prescription rate during intervention from November 2005 to May 2006	n.r.
Cals et al. 2010** Netherlands Two-armed randomised controlled intervention study	> 18 years First episode of lower RTI or rhinosinusitis (max. for 4 weeks)	T1 and T2: 129/129; 43.0/45.5 33 physicians (in 11 practices) on T1 and T2	IG: POCT for CRP measurement, CRP-dependent prescribing strategies: immediate or delayed prescribing or no prescribing CG: care as usual	Two data collection periods from November 2007 to April 2008: T1: after initial consultation T2: 28 days after initial consultation	No hospitalisations and deaths during the study
Linder et al. 2010 USA Two-armed cluster-randomised controlled intervention study	Consultation due to upper/lower acute RTI	Number of consultations due to RTI: T1: 8406/10082; 49/49 Physicians on T1: 258/315 in 27 “practice centres”	IG: “ARI Quality Dashboard” = CDSS Collection of patient data, documentation of diagnosis and therapy, presentation of therapy options with integrated CDSS Comparison of indiv. Abx prescription rate with national RTI-prescribing data Management of billing data CG: care as usual	Abx prescription rate right after initial consultation: T0: baseline T1: Abx prescription rate during intervention from November 2006 to August 2007	n.r.
Worrall et l. 2010 Canada Two-armed randomised controlled intervention study	> 18 years patients with acute upper RTI	T1: 75/74; n.r. Physicians on T1: 6	Arm 1: delayed prescribing with “normal” prescription Arm 2: delayed prescribing with post-dated prescription (48 h after initial consultation)	Abx prescription rate during 19 days after initial consultation: T1: n.r.	n.r.
Llor et al. 2011 Spain Two-armed randomised controlled intervention study	14–60 years patients with acute pharyngitis (≥ 1 censor criterion)	T1: 281/262; 31.8/31.5 Physicians on T1: 33/28 10 “primary care centres” in IG and CG	IG: RADT* (Strep A-Test) CG: care as usual	Abx prescription rate right after initial consultation: T1: January–May 2008	Side effects of AB therapy (gastrointestinal side effects, rash): I: 32 C: 8
McGinn et al. 2013 USA Two-armed cluster-randomised controlled intervention study	Patients > 18 years were included, if electronic health record detected keywords (=diagnoses, symptoms associated with pharyngitis or pneumonia)	T1: 586/398; 43/49 In total: 168 assistant physicians, specialists and specialised nurses in 2 primary care practices	IG: 1) 1-h training on Walsh score for streptococcal pharyngitis and Heckerling score for pneumonia 2) Video presentation of CDSS* and integration in practice software 3) Entry of keywords in practice software (on pharyngitis and pneumonia); pop-up function of CDSS* with following risk score calculation and corresponding recommendations CG: (Journal article about Walsh score for streptococcal pharyngitis and Heckerling score for pneumonia)	T1: Abx prescription rate right after initial consultation (additionally prescribed Abx 2 weeks after initial consultation) in November 1, 2010–October 31, 2011	Difference in emergency room visits between IG and CG: *p* > 0.99 Difference in follow-up treatment rate between IG and CG: *p* = 0.10
Hui Min Lee et al. 2016 SingaporeTwo-arm parallel group randomised controlled trial	Patients ≥ 21 years presenting with at least one of four symptoms (runny nose, blocked nose, cough or sore throat) for 7 days or less	T1: 457/457; 36/35 35 participating GPs from 24 clinics	IG: patients were educated on causes of upper respiratory tract infections CG: patients were educated on influenza vaccinations	Abx prescription rate after the initial consultation: T1: 8 working days in February 2015	n. r.

*Abx prescription rate antibiotic prescription rate, *CRP* C-reactive protein, *CDSS* clinical decision support systems, *CST* communication skills training, *CG* control group, *EHR* electronic health record, *IG* intervention group, *n*.*r*. not reported, *POCT* point-of-care-testing, *RADT* rapid antigen detection test, *RTI* respiratory tract infection

**Cals et al. 2009 and Cals et al. 2010: Antibiotic prescription data of the patient sample were captured during the index consultation (T1) and within 28 days after the index consultation. As T2 prescription rates include T1 prescription rates, this study is considered as a study with one post-intervention measurement

All cluster-randomised trials performed cluster-adjusted data analyses. The number of participating physicians ranged from 6 to 573. Most trials were conducted in Europe [13, 14, 20–22, 27, 28, 31], six trials in North America [15, 23–25, 30, 32] and one in Asia [33]. One trial was a multinational project carried out in six European countries (Belgium, Spain, Wales, England, Poland and the Netherlands) [26]. Published baseline data was available for seven trials [14, 20, 23, 26–29]. Eleven trials assessed the Abx prescription rate right after patients’ initial consultation [13, 20, 22, 23, 26–31, 33].

### Primary endpoints

Data on Abx prescription rates was collected directly by physicians [20, 21, 26, 27, 31], by pharmacists using faxed or mailed prescriptions [21, 32], by field researchers [33] or by electronic medical records [13, 15, 22–25, 28, 29]. Four trials used special documentation software [14, 23–25].

The time period for registration of Abx prescriptions ranged from right after the initial consultation up to 28 days after initial consultation.

Six trials assessed effectiveness of the intervention after a longer period of time, within 1 year [15, 29] or within 18 months after the intervention [30], after 1 year [13, 20] or after 3.5 years (Tables 3 and 4) [34].

### Description of participating physicians

Ten trials recruited primary care physicians in private practices [13, 14, 20–22, 26–29, 32], and seven trials recruited physicians from primary care clinics [15, 23–25, 30, 31, 33].

### Description of patient population

Patients ≥ 13 years with acute upper and lower RTIs were included. The average age of patients was similar across trials and ranged from 40 to 53 years. The number of registered consultations varied from 149 to 1,115,359 [28, 32].

### Description of interventions

#### Multifaceted interventions

Twelve RCTs used multifaceted interventions [13, 14, 20–26, 28–30]. Multifaceted interventions contain two or more components and address the different aspects of inadequate antibiotic prescribing. Due to the multifaceted interventions of the included trials, some of them are discussed repeatedly in the following subsections. For example, the factorial study design of Cals et al. allows reporting of the effect of the CST or POCT alone or combined [22].

#### Intervention elements addressing physicians

Twelve trials used interventions that addressed physicians [13, 14, 20–24, 26, 28–30, 32]. Four different types of interventions were evaluated:

1. A “classic” knowledge transfer approach using interactive seminars [13, 14, 21] and distribution of printed teaching and information materials [21, 23] as well as feedback on individual Abx prescription rates [13, 14, 21, 22, 24, 29]. Themes discussed included diagnosis-making and therapy of RTIs in accordance with guidelines as well as the challenge of increasing bacterial resistance.
2. A CST dealing with perceived pressure to prescribe where physicians learnt how to communicate with patients about their expectations on antibiotic prescribing and how to respond to patients’ concerns. In three trials, physicians were trained in seminars [20–22]. One trial provided an Internet-based CST [26].
3. Physicians were introduced to the concept of DP. This implies advising patients with low probability of bacterial RTI to use a prescription for antibiotics only in case symptoms do not resolve or get worse up to a pre-defined point in time. Cals et al. combined this strategy with C-reactive protein (CRP) POCT [30]. POCT are simple diagnostic tests and allow measuring CRP directly in the practice. CRP is an acute-phase protein with increasing plasma concentration during inflammatory processes. The measurement of CRP with a POCT has been proved accurate and can increase diagnostic certainty if combined with clinical examination—especially for identifying patients at high risk of pneumonia [35, 36]. Gjelstad et al. implemented additional software applications that asked physicians to specify whether the concept of DP was used and to document the number of days agreed to postpone antibiotic use [14]. Worrall et al. compared two DP procedures—one of them employing a ready-to-use prescription and the other applying a post-dated prescription usable only up to 48 h after initial consultation [32]. Gulliford implemented information about DP within a CDSS [28].
4. Electronic health records (EHR) asked physicians to justify their treatment decision if an antibiotic was ordered and provided alternative treatment interventions [29].

#### Intervention elements addressing patients

Five trials implemented interventions addressing patients [20, 23, 25, 28, 33]. Four trials used patient brochures with information about RTIs as adjunct [20, 23, 25, 28]. One trial used an additional waiting room poster addressing increasing bacterial resistance and prescribing pressure as one of the main reasons for inadequate prescribing [20]. Linder et al. and Gulliford et al. implemented documentation software with the possibility to print patient information leaflets [25, 28]. In the RCT of Linder et al., it remained unclear how many physicians used this possibility [25]. In the trial of Gulliford et al., the number of printed leaflets was low among the physicians with the highest utilisation of the CDSS (25 leaflets per 1000 consultations for RTI) [28]. In the trial of Hui Min Lee et al., patients in the IG were educated on the aetiology of upper RTIs by trained field researchers prior to the consultation [33].

#### Intervention elements addressing improved diagnosis-making

Twelve trials implemented interventions addressing diagnosis-making [13, 15, 22–31]. In one trial, the POCT was combined with a CST [22]. Another trial combined POCT with the strategy of DP [30]. Little et al. provided CRP-POCT training via the Internet [26]. Andreeva et al. used the CRP-POCT as a single intervention [27]. Bjerrum et al. employed a rapid antigen detection test for identifying group A streptococcal infections (RADT) in combination with feedback on personal prescribing rates [13]. Llor et al. used the RADT by itself [31]. The RADT is a fast pathogen identification test and can assist a physician in differentiating between a bacterial pharyngitis caused by group A streptococci or a viral infection. In combination with clinical scores such as the McIsaac score, it can raise diagnostic certainty and help to avoid unnecessary antibiotic prescriptions [37].

Six trials made use of CDSS [15, 23–25, 28, 29]. Linder et al. [24, 25] and McGinn et al. [15] provided assistance for estimating the likelihood of a bacterial RTI [24, 25] or a pneumonia/streptococcal pharyngitis [15]. Gulliford et al. provided evidence from research for antibiotic prescribing when a RTI was coded in an electronic medical record [28]. Andreeva et al. compared two different methods of diagnostic assistance: a computer-based system and a poster with a clinical algorithm [23]. Meeker et al. asked their participating physicians to justify their entered diagnosis and treatment [29].

### Effects of the intervention on Abx prescription rate (see Tables 1 and 2 and Figs. 3 and 4)

Twelve trials reported statistically significant lowered Abx prescription rates in the IGs compared to CGs [13–15, 20, 22, 23, 26–31]. In five RCTs. the Abx prescription rates could not be reduced significantly (see Tables 1 and 2) [21, 24, 25, 32, 33]. Using our definition for a clinically relevant reduction of prescription as criterion for efficacy, only six trials had a meaningful effect on Abx prescription rates [22, 23, 26, 27, 30, 31]. The effect of the interventions cannot be compared directly due to heterogeneous study designs.

**Fig. 3:**
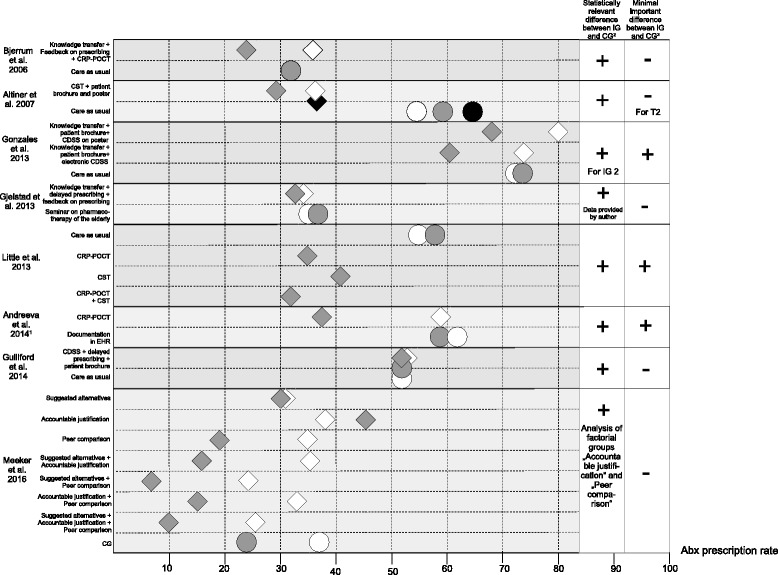
Abx prescription rates of trials with baseline and post-intervention measurements. Rhombus = intervention group = . Circle = control group = . White = T0 = pre-intervention measurement/baseline. Grey = T1 = post-intervention measurement. Black = T2 = optional post-intervention measurement. 1 Results for subgroup of 13 physicians. 2 Evaluated by study team. 3 According to our definition: For studies with baseline and post-intervention measurements, a difference in differences of 10 percentage points within study arms is regarded as minimal important change

**Fig. 4:**
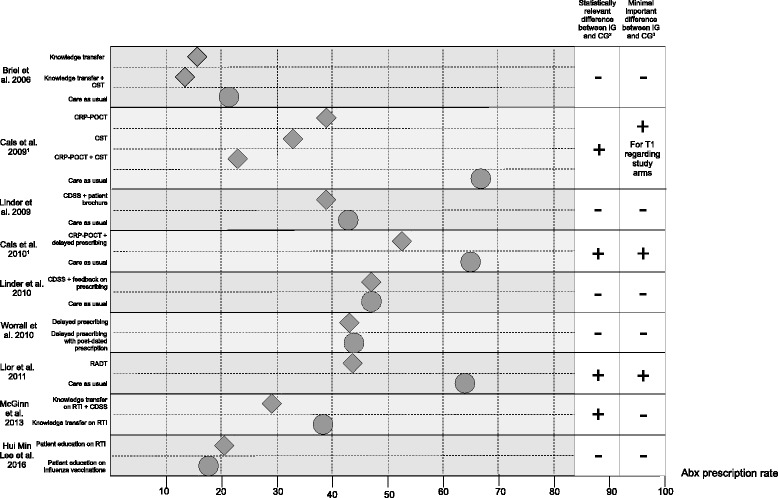
Abx prescription rates of trials with post-intervention measurements. Rhombus = intervention group = . Circle = control group = . Grey = T1 = post-intervention measurement. 1 Cals et al. 2009 and Cals et al. 2010: Antibiotic prescription data of the patient sample was captured during the index consultation (T1) and within 28 days after the index consultation (T2). Abx prescription rates for study arms are only available for T1. 2 Evaluated by study team. 3 According to our definition: For studies with post-intervention measurements, a difference of 10 percentage points between CG and IG is regarded as minimal important difference

#### Intervention effect of trials with baseline and post-intervention measurements

The Abx prescription rates of studies with baseline and post-intervention measurements are provided in Fig. 3.

In the trial of Bjerrum et al., the CG has in contrast to the IG no pre-intervention measurement [13]. All but one had an observation period ranging from measurement direct after the consultation up to 1 year [27]. The baseline prescription rates ranged from 24.4% [29] to 80% [23]. All studies found statistically significant results within and/or in-between study arms. The trial of Altiner et al. showed a large difference in baseline prescription rates, indicating the possibility of selection bias. Additionally, the initially observed effects after 6 weeks were not sustainable in this trial (see Fig. 3) [20]. Three studies reached a clinically relevant difference in differences greater than 10% [23, 26, 27]. Although, Gjelstad et al. observed a statistically significant reduction in prescribing rates, the overall effect of − 1.52% in the IG compared to + 1.7% in the CG is negligible [14]. Comparing baseline and post-intervention Abx prescription rates, changes within the IGs ranged from + 0.3 to − 23.3% [20, 26]. Changes within CGs ranged from + 10.1 to − 13.1% [20, 29].

#### Intervention effect of trials with post-intervention measurements

A total of nine studies reported on post-intervention Abx prescription rates (Fig. 4) [15, 21, 22, 24, 25, 30–33].

The reported prescription rates ranged from 13.5% [21] to 67% [22]. Only four of those trials observed a statistically significant reduction of Abx prescription rates [15, 22, 30, 31], but only three of them reached a difference between IG and CG exceeding 10% [22, 30, 31].

All three studies with a factorial design had a positive effect on reducing Abx prescription rates [26, 29, 30]. Studies with reported prescription rates lower than 20% had no significant reduction in Abx prescribing rates [21, 33].

Differences of + 2.9% to − 44% were observed in the IGs compared to CGs on post-intervention periods [22, 33] (see Table 2).

#### Effects of single-element interventions

Single-element interventions address one specific reason for inadequate prescribing. Nine RCTs implemented single-element interventions [15, 21, 22, 26, 27, 29, 31–33], three of them within a factorial study design [22, 26, 29].

All these RCTs contained interventions addressing either physicians [21, 22, 26, 29, 32], patients [33] or diagnosis-making [15, 22, 26, 27, 29, 31]. In the RCT by Worrall et al., DP with a post-dated prescription did not significantly reduce antibiotic use compared to usual DP [32]. Hui Min Lee et al. did not find significantly reduced Abx prescriptions by means of patient education on causes of upper RTIs compared to CG (20.6 vs. 17.7% in the CG, OR = 1.20, *p* = 0.313) [33]. The limited intervention in the RCT by Briel et al. could not significantly reduce Abx prescriptions [21].

Llor et al. implemented a more effective intervention: After initial consultation, the Abx prescription rate was 44% in the IG (*p* < 0.001) as compared to 64% in the CG due to RADTs [31].

McGinn et al. observed an Abx prescription rate of 29% in the IG and a rate of 38% in the CG (*p* = 0.008). Andreeva et al. registered a significant difference in antibiotic prescriptions within 2 weeks after initial consultation: 40.6% in the IG and 71.8% in the CG (*p* = 0.0001) [15].

In the trial by Cals et al., CRP-POCT reduced the Abx prescription rate by 13% compared to control (*p* < 0.01) [22]. Investigating long-term effects, after 3.5 years no effect was found. CST was able to reduce the Abx prescription rate, re-measured 28 days after consultation, by 25% (*p* < 0.001). After 3.5 years, patients in the CST group received significantly less antibiotic prescriptions for RTIs than in the CG (*p* < 0.02) [34].

Little et al. reduced the Abx prescription rate in the cumulative CST group (36 vs. 45%, OR = 0.69; 95% CI 0.54–0.87, *p* < 0.0001) and CRP POCT group (33 vs. 48%; OR = 0.54; 95% CI 0.42–0.69, *p* < 0.0001) compared in the control [26].

Meeker et al. implemented three single-element interventions (two of them focusing at physicians, one focusing at diagnosis-making) in a factorial study design: Intervention 1 “Suggested alternatives” did not significantly reduce Abx prescriptions (*p* = 0.66 for differences in trajectories). Intervention 2 “Accountable Justification” and intervention 3 “Peer comparison” registered significant differences in the rate of inappropriate antibiotic prescribing (*p* < 0.001) [29].

#### Effects of multifaceted interventions

Multifaceted interventions address different aspects of inadequate antibiotic prescribing—such as CST or POCT addressing prescribing pressure or diagnostic uncertainty. In most cases, multifaceted interventions contain intervention elements from at least two out of three “target groups”: physicians, patients or the process of diagnosis-making [13, 20–26, 28–30]. However, according to our definition, a multifaceted intervention can only focus at physicians or patients or diagnosis-making if different aspects are taken into account [14]: One RCT can just focus at physicians but with prescribing feedback and implementation of DP serving as a multifaceted intervention.

Interventions focusing on patients alone were mostly used as additional elements in multifaceted interventions [20, 23, 25, 28].

### Effects of multifaceted interventions addressing physicians, patients and the process of diagnosis-making

Two RCTs implemented interventions addressing all three “target groups”. Both trials could significantly reduce their Abx prescription rates [23, 28]. Gulliford et al. combined a CDSS with patient handouts and DP. The proportion of consultations with antibiotics prescribed declined marginally from 53 to 52% during 12 months after intervention, whereas it remained constant at 52% in the CG. The adjusted mean difference in antibiotic prescriptions was − 1.85% (*p* = 0.038) [28]. Gonzales et al. observed a reduction of 12% in the first IG (poster with clinical examination algorithm) and a reduction of 13% in the second group (CDSS). Both interventions were combined with patient handouts, feedback on prescribing and seminars for physicians. Compared to CG, both interventions were statistically significant (*p* = 0.003 or *p* = 0.01), but not between themselves (*p* = 0.67) [23].

### Effects of multifaceted interventions addressing physicians and the process of diagnosis-making

Five RCTs combined physician- and diagnosis-centred interventions [13, 22, 24, 26, 30], four of them reducing Abx prescription rates to a statistically significant extent [13, 22, 26, 30]: In their 2010 trial, Cals et al. reduced the Abx prescription rate by 12% (RR in the IG = 0.81, 95% CI 0.62–0.99) within a 28-day follow-up with the help of CRP POCT and DP [30]. Bjerrum et al. implemented CRP POCT, seminars for physicians and feedback on prescribing. The Abx prescription rate in the IG was 24% (CG 32%) 1 year after the intervention [13]. In the RCT by Little et al., the combination of CST and CRP-POCT led to a significant reduction of Abx prescriptions compared to CG (58 vs. 32%; *p* < 0.001) [26]. As the trial of Cals et al. was designed as a factorial trial, there was no testing for significance for the multifaceted intervention consisting of CRP POCT and CST [22]. The Abx prescription rate at index consultation was 23% (95% CI 11.6–34.6) and lower than in the CG (67%; 95% CI 53.9–79.5). Prescription rates for follow-up were not indicated. The RCT by Linder et al. from the year 2010 used CDSS and feedback on prescribing and found no difference between IG and CG [24].

### Effects of multifaceted interventions addressing patients and the process of diagnosis-making

The RCT by Linder et al. from 2009 tested patient handouts in combination with a CDSS. This intervention led to a non-significant reduction of 4% in the IG [25].

### Effects of multifaceted interventions addressing patients and physicians

In the trial by Altiner et al., the intervention contained patient brochures, a waiting room poster with information on RTI and a CST for physicians. The observed Abx prescription rate after 1 year was 36.7% in the IG compared to 64.8% in the CG. Yet, different baseline rates should be considered (36.4 vs. 54.7%) [20].

### Effects of multifaceted interventions focusing at physicians

Three RCTs used multifaceted interventions focusing at physicians [14, 21, 29]. Gjelstad et al. observed a reduction of 1.5% in the IG (*p* value = 0.027) [14]. The IG received feedback on prescribing, DP and seminars for physicians. However, they registered an increase of 1.7% in the CG (*p* value = 0.002). Continuing medical education groups with the corresponding prescription rates served as calculation basis instead of physicians with their individual prescribing rates.

In the trial of Briel et al., physician education alone reduced Abx prescription rates by 5.7%. In combination with CST, the reduction was increased to 7.9% compared to the CG. Both differences were not statistically significant [21]. The combination of interventions in the RCT by Meeker et al. did not result in statistically significant lowered inappropriate Abx prescription rates compared to CG [29].

### Relevance of intervention effects (see Table 6)

Table 6 shows the number of studies and intervention concepts with clinically relevant reductions of Abx prescription rates with regard to type of intervention. Six trials had a meaningful effect on Abx prescription rates [22, 23, 26, 27, 30, 31]. Three out of these six RCTs had a three-armed or factorial study design, therefore containing more than one intervention concept [22, 23, 26]. Altogether, our review contains 11 clinically relevant intervention concepts. The majority contained diagnosis-centred elements [22, 23, 26, 27, 30, 31], especially CRP POCT [22, 26, 27, 30].

POCT (CRP and RADT) and CST reduce effectively antibiotic prescriptions alone or in combination [22, 23, 26, 30]. One RCT combined CDSS with knowledge transfer and patient handouts effectively [23].

#### Clinically relevant single-element interventions

Six single-element intervention concepts could reduce Abx prescription to a clinically relevant extent [22, 26, 27, 31]. Four out of these six single-element interventions used diagnosis-centred interventions [26, 27, 31, 34]. All four tested POCT: Three measured the level of CRP [22, 26, 27], and one RCT used a RADT [31].

Two RCTs implemented the CST as an intervention focusing at physicians [22, 26].

#### Clinically relevant multifaceted interventions

Five intervention concepts led to a clinically relevant reduction [22, 23, 26, 30]. All interventions combined elements focusing at physicians and at the process of diagnosis-making [22, 26, 30]. Two intervention concepts had additional patient-centred elements [23]. The diagnosis-centred interventions contained CRP POCT [22, 26, 30] and CDSS [23].

### Secondary endpoints

Predefined secondary endpoints were patient-centred outcomes (e.g. reconsultation rate [21–23, 25–27, 29], patient satisfaction [21, 22, 30], patients’ views on RTIs [33] or days to recovery [22, 26, 27, 30, 31]), outcomes related to antibiotic prescribing (e.g. Abx prescriptions according to guidelines [24, 25, 31], class of prescribed antibiotic [13–15, 31], prescribed antibiotics for specific RTIs [13, 28]) or diagnostics (e. g. number of X-rays [27], number of RADTs [15]). Four RCTs did *not* find any significant difference in secondary endpoints between IG and CG [20–23]. One RCT did not provide any information on secondary endpoints [32].

#### Differences in patient-centred secondary outcomes

Four trials reported on differences in patient-centred secondary outcomes [26, 29, 30, 33]. Patient satisfaction was higher in the IG (76.3 vs. 63.2% of patients who were at least very satisfied, *p* = 0.03) [30]. Patient’s understanding of RTI improved (*p* < 0.001) [33], and the return visit rate within 30 days after a visit *without* antibiotic prescription was higher in the IG (1.41 vs. 0.43% in the CG, *p* < 0.001) [29]. Patients treated by physicians who had been trained in CST required more days for resolution of symptoms in the IG (adjusted risk ratio in the IG 0.83, *p* = 0.002) [26].

#### Differences in physician-centred secondary outcomes

The Abx prescription rate for patients with specific RTIs such as acute cough and bronchitis was significantly lower in the IG in the trial by Gulliford et al. (− 2.49%, 95% CI 0.15–4.83%, *p* = 0.030) [28]. Four RCTs reported on prescribed classes of antibiotics [13–15, 31] (see Table 5—intervention influence on prescribed antibiotic class). The trial by Llor et al. is omitted in this table because it did not distinguish between IG and CG [31]. Two of these four trials witnessed a significant increase of narrow-spectrum penicillins [13, 14], whereas the proportion of other antibiotic classes declined: In the trial by Gjelstad et al., the prescribing of tetracylines and macrolides decreased significantly in the IG. Simultaneously, the proportion of macrolides increased in the CG [14]. McGinn et al. observed significant differences only for the prescription rates for quinolones. The prescribing rate in the IG was 9.9% compared to 19.6% in the CG (*p* = 0.02) [15].

**Table 5:** Intervention influence on prescribed antibiotic class

Antibiotic prescription rates								
	Study arm^a^	Narrow-spectrum penicillins	Broad-spectrum penicillins	Cephalosporines	Macrolides	Quinolones	Tetracyclines	Other classes of antibiotics
Bjerrum et al. 2006 Spain Included diagnoses: upper/lower RTI (e.g. acute otitis media, tonsillitis, pharyngitis, laryngitis, sinusitis, COPD, pneumonia)	IG T0	0.6% (95% CI 0.0–2.3%)	60.7% (95% CI 55.1–66.1%)	4.4% (95% CI 2.4–7.3%)	17.9% (95% CI 13.9–22.6%)	6.3% (95% CI 3.9–9.5%)	0.3% (95% CI 0.0–1.7%)	9.8% (95% CI 6.7–13.6%)
	IG T1	7.9% (95% CI 5.3–11.2%)	65.3% (95% CI 60.0–70.3%)	0.9% (95% CI 0.2–2.5%)	7.0% (95% CI 4.5–10.2%)	7.0% (95% CI 4.5–10.2%)	0.0% (95% CI 0.0–1.1%)	11.9% (95% CI 8.7–15.9%)
	CG T1	1.0% (95% CI 0.4–2.0%)	70.2% (95% CI 66.7–73.6%)	4.7% (95% CI 3.2–6.5%)	10.2% (95% CI 8.9–12.6%)	6.5% (95% CI 4.9–8.6%)	0.1% (95% CI 0.0–0.8%)	7.3% (95% CI 5.5–9.5%)
Gjelstad et al. 2013 Norway Included diagnoses: acute upper/lower RTI (e.g. bronchitis, tonsillitis, pneumonia, sinusitis)	IG T0	43.8%	8.75%	n.s.	26.6%	n.s.	19.3%	1.23%
	IG T1	53.2% (*p* < 0.001)	8.71% (*p* = 0.948)	n.s.	22.9% (*p* = 0.003)	n.s.	13.6% (*p* < 0.001)	1.20% (*p* = 0.876)
	CG T0	43.5%	10.0%	n.s.	25.5%	n.s.	19.3%	1.43%
	CG T1	41.7% (*p* = 0.045)	9.78% (*p* = 0.682)	n.s.	27.8% (*p* = 0.006)	n.s.	19.2% (*p* = 0.821)	1.46% (*p* = 0.908)
McGinn et al. 2013 USA Included diagnoses: pharyngitis, pneumonia		Penicillins		Cephalosporines	Macrolides	Quinolones	Tetracyclines	Other classes of antibiotics
	IG T1	24.0%		1.2%	65.5%	9.9%	n.s.	n.s.
	CG T1	22.2% (*p* = 0.75)		1.3% (*p* = 0.82)	58.8% (*p* = 0.29)	19.6% (*p* = 0.02)	n.s.	n.s.

*RTI* respiratory tract infection, *n*.*s*. not specified

^a^*IG* intervention group, *CG* control group

Four RCTs categorised the prescribed antibiotics in appropriate or inappropriate prescriptions [24, 25, 31]. However, definitions for (in-) appropriate prescribing differed: Linder et al. and Meeker et al. defined antibiotic-appropriate (pneumonia, streptococcal pharyngitis, sinusitis, otitis media) and non-antibiotic-appropriate diagnoses (non-streptococcal pharyngitis, influenza, acute bronchitis, non-specific upper RTIs) [24, 25]. Llor et al. defined antibiotic prescribing for patients *without* group A streptococcal infections as well as missing antibiotic treatment for patients *with* group A streptococcal infections as inappropriate [31].

Linder et al. implemented a CDSS. When a CDSS was used, the rate of antibiotic prescriptions for diagnoses with appropriate antibiotic treatment was higher as in consultations *without* CDSS (88 vs. 59%, OR = 5.0; 95% CI = 2.9–8.6) [25]. The rate of non-appropriate antibiotics was lower when physicians used the CDSS (32 vs. 43%, *p* = 0.004) [24]. Physicians who made use of a CDSS prescribed less antibiotics in all consultations for RTI (42 vs. 50%, *p* = 0.02) [24]. Meeker et al. found reductions from 15.6 to 19.5% in seven IGs due to behavioural interventions alone or in combination [29] (see Table 1 for details).

In the trial by Llor et al., a RADT test was tested. A higher number of inappropriate antibiotics was observed in the CG (60 vs. 27% in the IG, *p* < 0.001) [31].

#### Differences in diagnosis-centred secondary outcomes

The RCT by Andreeva et al. observed a lower rate for chest radiography in the IG (55.4 vs. 76%, *p* = 0.004) [27].

## Discussion

### Summary of main results

This review updates and summarises current evidence of various interventions in primary care on reducing Abx prescriptions in patients ≥ 13 years for acute RTI. Twelve out of 17 included RCTs showed a statistically significant lower Abx prescription rate in the IG [13–15, 20, 22, 23, 26–31]. However, only six of them reported a clinically relevant reduction according to our definition [22, 23, 26, 27, 30, 31]. Due to the three-armed or factorial study design, these six RCTs contained 11 clinically relevant intervention concepts. Interventions focusing at physicians (CST) and at the process of diagnosis-making (CRP POCT, RADT, CDSS) were—alone or in combination—the most effective interventions. Observed reductions for RCTs with baseline ranged from 1.5 to 23.3% and cannot be compared directly due to heterogeneous baseline Abx prescription rates, study designs and settings [14, 26]. For studies with post-intervention measurements, the differences between IG and CG were between 2.9 and − 44% [22, 33]. Studies with reported prescription rates below 20% did not show significant reductions in Abx prescribing rates [21, 33]. Post-intervention observation periods ranged from 2 weeks up to 3.5 years. Conclusions on long-term sustainability of interventions cannot be drawn.

### Meaning of the results and comparison with existing literature

Our findings are in line with other systematic reviews, which reported mixed results regarding interventions to reduce antibiotics with either a larger or narrower spectrum of interventions and setting [7, 8, 10, 38]. We focused on RTIs in primary care excluding other infectious conditions and settings like emergency rooms, hospitals or public campaigns [39, 40].

We included RCTs with single-element [15, 22, 26, 27, 29, 31–33] and multifaceted interventions [13, 14, 20–26, 29, 30] focusing at diagnosis-making [13, 15, 22–31], at physicians [13, 14, 20–24, 26, 28–30, 32] or at patients [20, 23, 25, 28, 33]. Nine intervention concepts with meaningful effects on Abx prescription rates contained interventions addressing the process of diagnosis-making [22, 23, 26, 27, 30, 31], five of them in combination with interventions targeting physicians [22, 23, 26, 30]. In contrast to the systematic review of 2005, single-element interventions can be effective (Table 6) [22, 23, 26, 27, 30, 31]. Interventions addressing patients were less likely to reduce Abx prescriptions to a meaningful extent. POCT (CRP and RADT) and CST—alone or in combination—reduce effectively antibiotic prescriptions [22, 23, 26, 27, 30].

**Table 6:** Clinically relevant interventions

Study	Multifaceted intervention	Single-element intervention	Intervention focusing at diagnosis-making	Intervention focusing at physicians	Intervention focusing at patients	Details about the intervention
Cals et al. 2009	x		x	x		CST + CRP POCT
Cals et al. 2009		x		x		CST
Cals et al. 2009		x	x			CRP POCT
Cals et al. 2010	x		x	x		CRP POCT + DP
Llor et al. 2011		x				RADT
Gonzales et al. 2013	x		x	x	x	Software for CDSS + knowledge transfer + feedback on prescribing + patient handouts
Gonzales et al. 2013	x		x	x	x	CDSS + knowledge transfer + feedback on prescribing + patient handouts
Little et al. 2013	x		x	x		CST + CRP POCT
Little et al. 2013		x		x		CST
Little et al. 2013		x	x			CRP POCT
Andreeva et al. 2014		x	x			CRP POCT

*CDSS* clinical decision support system, *CRP* C-reactive protein, *CST* communication skills training, *DP* delayed prescribing, *POCT* point-of-care test, *RADT* rapid antigen detection test for group A streptococci

### Differences in Abx prescription rates and effect sizes

We observed large differences in Abx prescription rates between countries ranging from 13.5 and 80% and within a country [21, 23]. There were five trials from the USA [15, 23–25, 29] where pre-intervention Abx prescription rates ranged from 24 to 80% [23, 29] and post-intervention Abx prescription rates varied from 29 to 47% [15, 24, 25]. There were two studies from the Netherlands with post-intervention Abx prescription rates ranging from 23 to 65% [22, 30]. Two studies from Spain showed post-intervention Abx prescription rates between 24 and 64% [13, 31]. Pre-intervention Abx prescription rates in the trial of Bjerrum et al. were only available for the IG [13]. These large variations within and between countries limit the generalisability of the findings and indicate high possibility of selection bias and regional factors affecting Abx prescription rates.

Five included trials did not demonstrate any reduced Abx prescription rates [21, 24, 25, 32, 33]. Possible reasons for lack of success were possible selection bias in the recruitment of physicians who were already low prescribers [21, 33], low intervention uptake or insufficient implementation [24, 25] as well as lack of power due to low number of participating physicians and patients [32]. We consider five trials reporting statistically significant results as ineffective [14, 15, 20, 28, 29]. Gulliford et al. used a CDSS in a large sample of family practices and reported a small difference of 1.85% (95% CI − 0.1 to 3.59) [28]. IG and CG had a similar baseline prescription rate of roughly 50% reflecting overprescribing. The reported statistical significance of the small observed effect is due to the large sample size and cannot be regarded as efficient (difference in differences: − 1%).

Altiner et al. implemented CST, patient brochures and a waiting room poster in the IG [20]. Despite a large difference in baseline prescription rates and increased Abx prescription rates in the IG (+ 0.3%) and CG (+ 10.1%) within 1 year after baseline, this trial reported statistically relevant reductions after adjusting for seasonal effects and confounding variables such as severity of disease (IG: adjusted OR = 0.72, 95% CI 0.54–0.97, *p* = 0.028; CG: adjusted OR = 1.31, 95% CI 1.01–1.71, *p* = 0.044). This reduction does not satisfy our conditions for a meaningful change (difference in differences − 9.8%).

In the trial of Gjelstad et al., the combination of knowledge transfer, DP and feedback on prescribing resulted in a small difference of − 4.1% between IG and CG [14]. Due to the large sample size, this trial reported statistically relevant reductions but cannot be regarded as a meaningful change (difference in differences + 0.2%).

Meeker et al. investigated three interventions separately and in combination within a factorial study design [29]. Although two of the interventions showed a statistically significant and impressive reduction of Abx prescriptions of 17%, this has to be interpreted given that a reduction of 13% in Abx prescriptions was also observed in the CG. An explanation for a reduction without intervention was not given, beside Hawthorne effect. This observation points out the importance of an independent CG and pre- and post-intervention measurements of prescribing rates. Therefore, all trials shown in Fig. 4 lacking pre-intervention measurements have to be interpreted cautiously. For example, McGinn et al. reported a trial with a difference of 9% in Abx prescription rates compared to the CG, just below our arbitrary threshold for minimal significance of 10% [15]. However, for both groups, no baseline prescription rates are available.

Our review suggests that, in countries with relatively low prescription rates like Germany or the Netherlands, CST seems to be the key element for successful interventions. In contrast, interventions focusing on making a diagnosis in terms of POCT and CDSS showed relevant reductions in high-prescribing countries (e.g. Spain, USA, Russia) [13, 23, 27]. The role of electronic decision support systems remains unclear.

### Secondary outcomes

This review also adds to our knowledge that interventions aiming at reducing inappropriate antibiotic prescribing can have a positive effect on the prescribing quality. In a number of trials, the number of narrow-spectrum penicillins increased [13, 14], whereas the proportion of broad-spectrum antibiotics declined [13–15], although the main focus of the interventions was to *not* prescribe. Effects on patients’ satisfaction were reported in three trials [21, 22, 30]. Only one trial reported a significantly higher proportion of patients satisfied with care in the IG due to CRP-POCT (*p* = 0.03).

### Limitations of the review

Our systematic literature search was limited to few databases and hand search of references due to lack of access to other databases and funding. Additionally, publication bias and the possible exclusion of some foreign language trials have to be acknowledged. Although we cannot exclude that we have missed few trials, we believe this would not have changed our conclusions or allowed summary statistics given the heterogeneity of the designs and outcome measures.

### Limitations of trials included

All included RCTs differed in study design, data collection and time points of measurement, trial quality and baseline prescribing rates. Reporting of trial data was often poor due to missing *p* values, confidence intervals, absolute number of prescriptions and/or baseline data (Tables 1 and 2). Trials with high risk of bias may have led to a too positive interpretation of reported results. The heterogeneity of trial designs and outcome measurements made it impossible to pool trial data or compare effect sizes (e.g. using Cohen’s *d*) between trials. Alternatively, we have summarised the baseline and post-intervention Abx prescription rates in Figs. 3 and 4 to illustrate the differences and heterogeneity in between trials. There is no consensus about the effect size on Abx prescription rates considered as minimal important change. Our arbitrary assumptions considering an absolute 10% change as minimal important is based on the impression gained from these figures. The majority of trials did not adjust or balance seasonal effects (winter vs. summer), possibly affecting Abx prescription rates.

### Conclusions and implications for research

CST and POCT alone or in combination have the potential to reduce antibiotic prescriptions for RTIs. Electronic decision support tools showed only mixed results. Eleven out of 17 trials were not successful in reducing Abx prescription rates according to our definition of minimal important change [13–15, 20, 21, 24, 25, 28, 29, 32, 33]. However, six of them reported a statistically significant reduction [13–15, 20, 28, 29]. Trials with low initial Abx prescription rates were less likely to be successful. Despite a number of noteworthy current studies, the generated evidence remains disappointingly limited. Only moderate evidence which interventional strategies are successful and how these findings could be generalised beyond the actual setting and the observational period of the trial exist.

We conclude that there is a need to develop a consensus for designing and reporting of trials aiming to reduce inappropriate Abx prescriptions in the near future. It should address (among others) the measurement of pre-intervention prescribing rates, adjustment for seasonal and temporal trends, (minimal) follow-up time, data analysis and reporting.

## Abbreviations

95% CI: 95% confidence interval
Abx prescription rate: Antibiotic prescription rate
CDSS: Clinical decision support system
CG: Control group
CRP: C-reactive protein
CST: Communication skills training
DP: Delayed prescribing
EHR: Electronic health record
IG: Intervention group
OR: Odds ratio
POCT: Point-of-care testing
RADT: Rapid antigen detection test
RCT: Randomised controlled trial
RR: Relative risk
RTI: Respiratory tract infection

## References

[1] Holmes WF, Macfarlane JT, Macfarlane RM, Hubbard R (2001). Symptoms, signs, and prescribing for acute lower respiratory tract illness. Br J Gen Pract.

[2] Spinks A, Glasziou PP, Del Mar CB. Antibiotics for sore throat. Cochrane Database Syst Rev. 2013;11:CD000023. 10.1002/14651858.CD000023.pub4 PMC6457983 24190439

[3] Roca I, Akova M, Baquero F, Carlet J, Cavaleri M, Coenen S (2015). The global threat of antimicrobial resistance: science for intervention. New Microbes New Infect.

[4] Sabuncu E, David J, Bernède-Bauduin C, Pépin S, Leroy M, Boëlle P-Y (2009). Significant reduction of antibiotic use in the community after a nationwide campaign in France, 2002–2007. PLoS Med.

[5] Rautakorpi U-M, Huikko S, Honkanen P, wKlaukka T, Mäkelä M, Palva E, et al. The Antimicrobial Treatment Strategies (MIKSTRA) Program: a 5-year follow-up of infection-specific antibiotic use in primary health care and the effect of implementation of treatment guidelines. Clin Infect Dis 2006; 42:1221–1230. 10.1086/503036 16586379

[6] Welschen I, Kuyvenhoven MM, Hoes AW, Verheij TJM (2004). Effectiveness of a multiple intervention to reduce antibiotic prescribing for respiratory tract symptoms in primary care: randomised controlled trial. BMJ.

[7] Coxeter P, Del Mar CB, McGregor L, Beller EM, Hoffmann TC. Interventions to facilitate shared decision making to address antibiotic use for acute respiratory infections in primary care. Cochrane Database Syst Rev. 2015;11:CD010907. 10.1002/14651858.CD010907.pub2 PMC6464273 26560888

[8] Drekonja DM, Filice GA, Greer N, Olson A, MacDonald R, Rutks I (2015). Antimicrobial stewardship in outpatient settings: a systematic review. Infect Control Hosp Epidemiol.

[9] World Health Organization (2015). Global action plan on antimicrobial resistance.

[10] Arnold SR, Straus SE. Interventions to improve antibiotic prescribing practices in ambulatory care. Cochrane Database Syst Rev. 2005;4:CD003539. 10.1002/14651858.CD003539.pub2 PMC7003679 16235325

[11] Moher D, Liberati A, Tetzlaff J, Altman DG (2009). Preferred Reporting Items for Systematic Reviews and Meta-Analyses: the PRISMA Statement. J Clin Epidemiol.

[12] Tates K, Meeuwesen L (2001). Doctor–parent–child communication. A (re)view of the literature. Soc Sci Med.

[13] Bjerrum L, Cots JM, Llor C, Molist N, Munck A (2006). Effect of intervention promoting a reduction in antibiotic prescribing by improvement of diagnostic procedures: a prospective, before and after study in general practice. Eur J Clin Pharmacol.

[14] Gjelstad S, Høye S, Straand J, Brekke M, Dalen I, Lindbæk M (2013). Improving antibiotic prescribing in acute respiratory tract infections: cluster randomised trial from Norwegian general practice (prescription peer academic detailing (Rx-PAD) study). BMJ.

[15] McGinn TG, McCullagh L, Kannry J, Knaus M, Sofianou A, Wisnivesky JP (2013). Efficacy of an evidence-based clinical decision support in primary care practices: a randomized clinical trial. JAMA Intern Med.

[16] Higgins JPT, Altman DG, Gøtzsche PC, Jüni P, Moher D, Oxman AD (2011). The Cochrane Collaboration’s tool for assessing risk of bias in randomised trials. BMJ.

[17] Cohen J (1988). Statistical power analysis for the behavioral sciences.

[18] Manager HCTPCT. Antibiotic use in outpatient settings [Internet]. [cited 2017 Nov 1]. Available from: http://pew.org/1N89p4C.

[19] de With K, Schröder H, Meyer E. Antibiotikaanwendung in Deutschland im europäischen Vergleich. Dtsch Med Wochenschr. 2004;129:1987–92.

[20] Altiner A, Brockmann S, Sielk M, Wilm S, Wegscheider K, Abholz H-H (2007). Reducing antibiotic prescriptions for acute cough by motivating GPs to change their attitudes to communication and empowering patients: a cluster-randomized intervention study. J Antimicrob Chemother.

[21] Briel M, Langewitz W, Tschudi P, Young J, Hugenschmidt C, Bucher HC (2006). Communication training and antibiotic use in acute respiratory tract infections. A cluster randomised controlled trial in general practice. Swiss Med Wkly.

[22] Cals JWL, Butler CC, Hopstaken RM, Hood K, Dinant G-J (2009). Effect of point of care testing for C reactive protein and training in communication skills on antibiotic use in lower respiratory tract infections: cluster randomised trial. BMJ.

[23] Gonzales R, Anderer T, McCulloch CE, Maselli JH, Bloom FJ, Graf TR (2013). A cluster-randomized trial of decision support strategies for reducing antibiotic use for acute bronchitis. JAMA Intern Med.

[24] Linder JA, Schnipper JL, Tsurikova R, Yu T, Volk LA, Melnikas AJ, et al. Electronic health record feedback to improve antibiotic prescribing for acute respiratory infections. Am J Manag Care [Internet]. 2010;16. [cited 2017 Mar 14]. Available from: http://www.ajmc.com/journals/supplement/2010/ajmc_10dec_hit/ajmc_10hitdeclinder_exclu_e311 21322301

[25] Linder JA, Schnipper JL, Tsurikova R, Yu T, Volk LA, Melnikas AJ (2009). Documentation-based clinical decision support to improve antibiotic prescribing for acute respiratory infections in primary care: a cluster randomised controlled trial. Inform Prim Care.

[26] Little P, Stuart B, Francis N, Douglas E, Tonkin-Crine S, Anthierens S (2013). Effects of internet-based training on antibiotic prescribing rates for acute respiratory-tract infections: a multinational, cluster, randomised, factorial, controlled trial. Lancet Lond Engl.

[27] Andreeva E, Melbye H (2014). Usefulness of C-reactive protein testing in acute cough/respiratory tract infection: an open cluster-randomized clinical trial with C-reactive protein testing in the intervention group. BMC Fam Pract.

[28] Gulliford MC, van Staa T, Dregan A, McDermott L, McCann G, Ashworth M (2014). Electronic health records for intervention research: a cluster randomized trial to reduce antibiotic prescribing in primary care (eCRT study). Ann Fam Med.

[29] Meeker D, Linder JA, Fox CR, Friedberg MW, Persell SD, Goldstein NJ (2016). Effect of behavioral interventions on inappropriate antibiotic prescribing among primary care practices: a randomized clinical trial. JAMA.

[30] Cals JWL, Schot MJC, de Jong SAM, Dinant G-J, Hopstaken RM (2010). Point-of-care C-reactive protein testing and antibiotic prescribing for respiratory tract infections: a randomized controlled trial. Ann Fam Med.

[31] Llor C, Madurell J, Balagué M, Gómez M, Cots JM (2011). Impact of rapid antigen detection testing on antibiotic prescription in acute pharyngitis in adults. A multicentric randomized controlled trial. Br J Gen Pract.

[32] Worrall G, Kettle A, Graham W, Hutchinson J (2010). Postdated versus usual delayed antibiotic prescriptions in primary care: reduction in antibiotic use for acute respiratory infections?. Can Fam Physician Med Fam Can.

[33] Hui Min Lee M, Shaw Teng Pan D, Huixin Huang J, I-Cheng Chen M, Hui Goh E, Jiang L. Efficacy of a patient-based health education intervention in reducing antibiotic use for acute upper respiratory tract infections in the private sector primary care setting in Singapore. Antimicrob Agents Chemother. 2017;61(5). 10.1128/AAC.02257-16 PMC5404603 28193663

[34] Cals JWL, de Bock L, Beckers P-JHW, Francis NA, Hopstaken RM, Hood K (2013). Enhanced communication skills and C-reactive protein point-of-care testing for respiratory tract infection: 3.5-year follow-up of a cluster randomized trial. Ann Fam Med.

[35] Chalmers JD, Singanayagam A, Hill AT (2008). C-reactive protein is an independent predictor of severity in community-acquired pneumonia. Am J Med.

[36] Hopstaken RM, Muris JW, Knottnerus JA, Kester AD, Rinkens PE, Dinant GJ (2003). Contributions of symptoms, signs, erythrocyte sedimentation rate, and C-reactive protein to a diagnosis of pneumonia in acute lower respiratory tract infection. Br J Gen Pract.

[37] Tanz RR, Gerber MA, Kabat W, Rippe J, Seshadri R, Shulman ST (2009). Performance of a rapid antigen-detection test and throat culture in community pediatric offices: implications for management of pharyngitis. Pediatrics.

[38] Spurling GKP, Del Mar CB, Dooley L, Foxlee R, Farley R. Delayed antibiotics for respiratory infections. Cochrane Database Syst Rev. 2013;4:CD004417. 10.1002/14651858.CD004417.pub4 23633320

[39] Rubin MA, Bateman K, Alder S, Donnelly S, Stoddard GJ, Samore MH (2005). A multifaceted intervention to improve antimicrobial prescribing for upper respiratory tract infections in a small rural community. Clin Infect Dis.

[40] Mölstad S, Erntell M, Hanberger H, Melander E, Norman C, Skoog G (2008). Sustained reduction of antibiotic use and low bacterial resistance: 10-year follow-up of the Swedish Strama programme. Lancet Infect Dis.

